# Concordance of copy number abnormality detection using SNP arrays and Multiplex Ligation-dependent Probe Amplification (MLPA) in acute lymphoblastic leukaemia

**DOI:** 10.1038/s41598-019-56972-0

**Published:** 2020-01-08

**Authors:** Matthew Bashton, Robin Hollis, Sarra Ryan, Claire J. Schwab, John Moppett, Christine J. Harrison, Anthony V. Moorman, Amir Enshaei

**Affiliations:** 1 Translational and Clinical Research Institute, Faculty of Medical Science, Centre for Cancer, Newcastle University, Newcastle upon Tyne, NE1 7RU UK; 2 Department of Haematology, Royal Hospital for Sick Children, Bristol, BS2 8BJ UK; 3 Department of Applied Sciences, Faculty of Health and Life Sciences, Northumbria University, Newcastle upon Tyne, NE1 8ST UK

**Keywords:** Paediatric cancer, Risk factors

## Abstract

In acute lymphoblastic leukaemia, MLPA has been used in research studies to identify clinically relevant copy number abnormality (CNA) profiles. However, in diagnostic settings other techniques are often employed. We assess whether equivalent CNA profiles are called using SNP arrays, ensuring platform independence. We demonstrate concordance between SNP6.0 and MLPA CNA calling on 143 leukaemia samples from two UK trials; comparing 1,287 calls within eight genes and a region. The techniques are 99% concordant using manually augmented calling, and 98% concordant using an automated pipeline. We classify these discordant calls and examine reasons for discordance. In nine cases the circular binary segmentation (CBS) algorithm failed to detect focal abnormalities or those flanking gaps in *IKZF1* probe coverage. Eight cases were discordant due to probe design differences, with focal abnormalities detectable using one technique not observable by the other. Risk classification using manually augmented array calling resulted in four out of 143 patients being assigned to a different CNA risk group and eight patients using the automated pipeline. We conclude that MLPA defined CNA profiles can be accurately mirrored by SNP6.0 or similar array platforms. Automated calling using the CBS algorithm proved successful, except for *IKZF1* which should be manually inspected.

## Introduction

B-cell precursor acute lymphoblastic leukaemia (B-ALL) arises from the accumulation of immature cells within the bone marrow and blood, and is characterised by key chromosomal and genetic abnormalities^1^. Accurate risk stratification of B-ALL is essential for assignment of patients to appropriate treatment regimens, balancing the efficacy of treatment with the cytotoxic nature of the chemotherapeutic agents. B-ALL cases are risk stratified into high, low and intermediate risk, on the basis of primary genomic abnormalities, which are routinely detected by techniques such as karyotyping and fluorescence *in situ* hybridization (FISH)^2^. However, approximately 25% of patients with B-ALL lack the common primary chromosomal abnormalities. This group, termed B-other-ALL, are classed as intermediate risk, as indicated in the paediatric treatment trial, UKALL97/99^3^. Within this subgroup, there is a clinical need to identify genetic biomarkers to enable risk stratification. Current risk stratification algorithms use a combination of age, white cell count (WCC), minimal residual disease (MRD) and chromosomal abnormalities. Novel risk stratification algorithms have been developed that incorporate copy number abnormalities (CNAs), including gains, amplifications, losses, and deletions of specific genes or genomic regions, including sets of genes^4^. These algorithms will be used alongside established risk factors to stratify patients in upcoming European trials for ALL; AIEOP-BFM ALL 2017^5^ and ALLTogether^6^. Both the UKALL-CNA^4^ profile and *IKZF1*^plus^ ^7^ were developed using copy number data generated by Multiplex Ligation-Depend Probe Amplification (MLPA). MLPA is a multiplexed PCR assay that uses probes specific for exons within genes of interest, enabling the relative copy number of each covered exon to be reported as a ratio against other probe(s) within reference regions. In addition, MLPA has been used to assist the molecular diagnosis of a wide variety of diseases^8^. SNP arrays are also used for detection of CNAs with several advantages: detection of CNAs involving all genes rather than a defined set, other clinically relevant events such as aneuploidy, ploidy changes or partial chromosomal imbalances can also be detected. From a clinical diagnostic laboratory perspective, SNP arrays provide a useful real-time technique, they are genome wide and not disease-specific such as most MLPA kits. Thus, many diagnostic laboratories frequently have SNP array platforms in place for other cancer work or constitutional genetics. While previous studies have compared MLPA and SNP array copy number concordance, they have either focussed on single genes or have been applied to the analysis of germline DNA; neither of which are relevant to validation of the UKALL-CNA classifier. In B-ALL specifically, Dörge *et al*. investigated concordance among 25 ALL patients for *IKZF1* abnormalities. They found 23 known *IKZF1* deletions detected by SNP6.0 array also to be detectable by MLPA, with minor differences at exon level due to probe positioning^9^. In addition, Zanardo *et al*. investigated a cohort of 93 patients with developmental and congenital abnormalities, using MLPA and SNP arrays and showed a 98% concordance between the two techniques^10^. To our knowledge, there has been no large-scale systematic study investigating the comparative performance of CNA detection using SNP array and MLPA techniques for an established set of key prognostic genes and genomic regions in leukaemia.

It is important to validate biomarkers using different techniques as well as different patient cohorts. The UKALL-CNA and the *IKZF1*^plus^ profiles have been validated across different patient populations. In this study, we validate the use of SNP arrays for detection of key CNAs, which underpin these two copy number profiles in ALL. In addition, we sought to compare the performance of the two techniques in order to assess both the concordance of SNP6.0 arrays and MLPA, as well as the ability of automated SNP array analysis to call CNAs.

## Methods

### Cohort and classification criteria

Patients were diagnosed with B-cell precursor ALL by standard flow-cytometric criteria and were treated on Medical Research Council (MRC) ALL97/99 (1997–2002) or United Kingdom UKALL2003 (2003–2011). Full details of these treatment regimens have been previously published^11,12^. Local ethical committee approval was obtained for ALL97 by individual treatment centres, whereas approval for UKALL2003 was obtained from the Scottish Multi-Centre Research Ethics Committee. Informed consent was given by parents and patients in accordance with the Declaration of Helsinki. Briefly, in ALL99 and UKALL2003, patients were assigned to regimen A or B based on whether they were National Cancer Institute (NCI) standard (<10 years old and WCC < 50 × 10^9^/L) or high risk (≥10 years old or WCC ≥ 50 × 10^9^/L), respectively.

Cytogenetic and FISH testing was performed on pre-treatment bone marrow samples by member laboratories of the UK Cancer Cytogenetics Group or centrally by the Leukaemia Research Cytogenetics Group, and results were reported using established nomenclature and definitions^3^. Patients with absence of the following chromosomal abnormalities: *ETV6-RUNX1*, high hyperdiploidy (51–65 chromosomes), *BCR-ABL1*, *KMT2A/MLL* rearrangements, near haploidy (<30 chromosomes), low hypodiploidy (30–39 chromosomes), intrachromosomal amplification of chromosome 21 (iAMP21) or *TCF3*-*HLF*; were classified as B-other-ALL and were included in this study.

### Array normalisation and copy number calling

DNA was hybridised to Affymetrix SNP6.0 arrays by AROS Applied Biotechnology A/S, Denmark. Data from array hybridisation was received as CEL files. PennCNV-Affy^13^ was used to perform quantile normalisation and calculate Log *R* ratio (LRR) data using HapMap reference clusters. This enabled all 1.8 million probes on a SNP6.0 array to be used; both copy number and SNP probes. The LRR data was GC content corrected for hg19 using PennCNV^14^. LRR data was then loaded in the R statistical computing environment^15^ and copy number segmentation was performed using the binary segmentation algorithm (CBS) implemented in the DNACopy^16,17^ package in Bioconductor^18^. An overview of the copy number pipeline is given in Supplementary Fig. S1. Full details of the automated copy number calling are given in the on-line supplemental methods sections.

### MLPA

MLPA was performed on DNA extracted directly from pre-treatment bone marrow samples, all samples had >90% blasts, sample handling and processing of cells was as previously described in Schwab *et al*.^19^. The same DNA source was used for MLPA and SNP6.0. The SALSA MLPA kit P335 (MRC Holland, Amsterdam, The Netherlands), which includes probes for *IKZF1*, *CDKN2A/B*, *PAX5*, *EBF1*, *ETV6*, *BTG1*, *RB1*, and the PAR1 region, was used to identify CNAs involving these genes^20^. Previous studies have demonstrated that MLPA can accurately detect deletions within each of these genes when present in 20% or more cells^19,20^. A detailed description and breakdown of each CNA and the correlation with specific chromosomal abnormalities for all the patients within these two cohorts has been previously published^20^. MLPA data analysis methodology and peak ratio values used to make MLPA calls are as previously described in Schwab *et al*.^19^.

### Copy number call comparison

Discordant calls between MLPA and the automated array pipeline were extracted from both datasets, each discordant call was manually inspected by three independent reviewers (MB, RH, AE) by visualising the LRR data for the gene of interest. This approach also permitted manual calling of focal events that were discernible by eye in the LRR plots but missed using the CBS algorithm. Joint manual review was undertaken to determine the nature and likely cause of discordance.

For the purpose of this study, we have considered losses/deletions and gains/amplifications, as the same event owing to the use of different copy number thresholds and sensitivities between SNP6.0 and MLPA. Additionally, as no formal definition or threshold for copy number gain versus amplification and similarly loss versus deletion exist, we have chosen to aggregate these two pairs of events in to either gain/amplification or loss/deletion.

Details of MLPA probe location determination methodology and representativeness of our cohort is given in the on-line supplemental methods section.

## Results

### Concordance of copy number abnormality calls

We compared the copy number calls from MLPA and our SNP array pipeline for 143 diagnostic samples from B-other-ALL patients treated on ALL97–99 or UKALL2003. The demographics of these patients are detailed in Supplementary Tables S1 and S2. Specifically, we compared the genes included in the P335-MLPA kit used in the UKALL-CNA classifier: *IKZF1*, *CDKN2A/B*, *PAX5*, *EBF1*, *ETV6*, *BTG1*, *RB1*, and the PAR1 region (*CRLF2/CSF2RA/IL3RA*)^4^.

**Figure 1:**
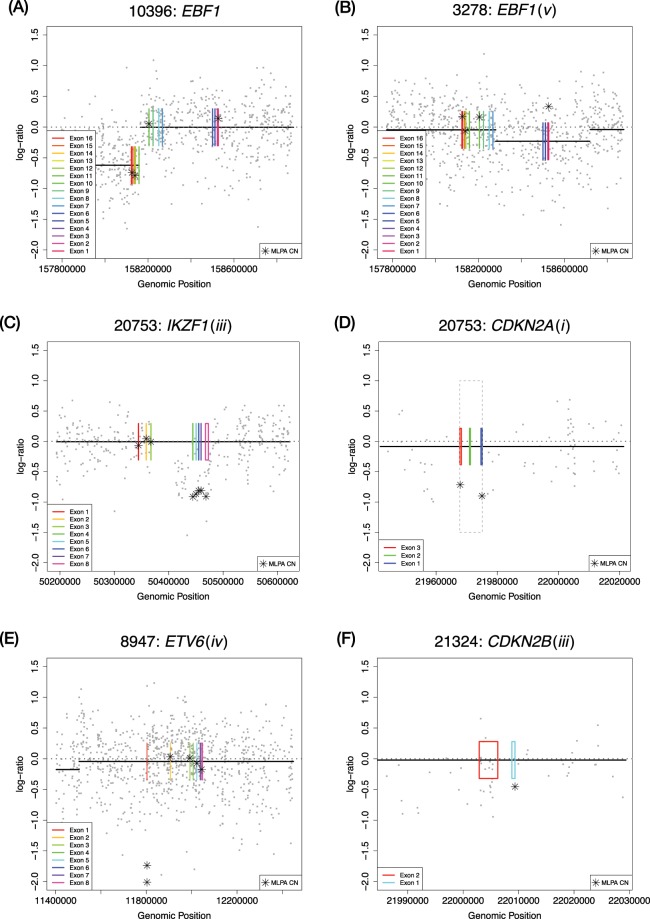
Examples of concordant and discordant calls by the SNP6.0 pipeline and MLPA. Patient ID and gene name, along with disagreement category in parenthesises are shown in the title of each plot. Individual SNP6.0 data points of Log *R* Ratio (LRR) are shown in grey, copy number calls by the binary segmentation algorithm are shown as black lines, with the copy normal LRR value of zero shown as a grey dotted line. Exons from genes are plotted as plotted boxes in rainbow colour, and MLPA data points converted into the LRR scale are shown as stars. (**A**) Shows a concordant Abnormal-Abnormal *EBF1* call. (**B**) Shows an example of a case in which limited coverage by MLPA misses a focal abnormality in *EBF1* exons 1 to 6, here the sole MLPA probe reports a slight gain but without further probes until exons 10, 14, and 16, this focal abnormality detected by SNP6.0 is missed in MLPA. (**C**) A highly focal abnormality occurring in *IKZF1* which was manually observed but the binary segmentation (CBS) algorithm failed to identify. The abnormality is very focal and flanks a region of the *IKZF1* gene where there is no probe coverage between *IKZF1* exons 3 and 4 (“*IKZF1* hole”). (**D**) A highly focal loss within *CDKN2A* is picked up by MLPA but is undetected by SNP6.0 array as no probes lie within *CDKN2A*. The grey dotted box shows the extents of *CDKN2A* in which no SNP6.0 LRR data points can be found. (**E**) An example of a case in which the two techniques disagree (discordance category iv). MLPA shows a deletion in two MLPA probes within exon 1 of *ETV6*, no concordant deflection of the SNP6.0 LRR scatter around the copy number normal log_2_ value of zero is observed. (**F**) Observation from a single MLPA probe which covers *CDKN2B* and indicates a loss, no concordant shifts can be seen in the LRR values of many SNP6.0 probes.

Using the value of copy returned by the CBS algorithm, we found 98% of the calls to be concordant between the SNP6.0 pipeline and MLPA, with 25 calls discordant out of a total of 1,287 (Table 1). Manual event calling by visual inspection of the plotted LRR data for cases which the CBS algorithm missed (nine cases) improved concordance to 99% reducing the number of discordant calls to 16 (Table 1). Discordant calls were classified into one of five categories: (i) no SNP array probes covering the abnormality – meaning that due to the array design it was impossible to make a call (*n* = 5). (ii) MLPA result was informed by a single MLPA probe (*n* = 2). (iii) The CBS algorithm failed to pick up a small focal abnormality, which could be called visually by inspection of the LRR data, such that they would be concordant with the MLPA calls (*n* = 9). (iv) The two techniques disagreed despite sufficient probe coverage (*n* = 6). (v) SNP6.0 detected an abnormality which could not be detected by MLPA due to lack of probe coverage (*n* = 3). Table 2 shows the discordant calls on a case-by-case basis, using both the manually augmented and fully automated CBS calling methods. Figure 1 shows examples of typical concordant and discordant profiles for each of the five categories.

**Table 1:** Concordance between SNP array and MLPA data.

		*IKZF1*			*ETV6*			*CDKN2A*	
		Normal	Abnormal		Normal	Abnormal		Normal	Abnormal
	**MLPA**								
**SNP**	Normal	113	1 iv	Normal	120	1 iv	Normal	83	3 i
	Abnormal	0	21, 8 iii	Abnormal	0	22	Abnormal	0	57
		***CDKN2B***			***RB1***			***BTG1***	
		Normal	Abnormal		Normal	Abnormal		Normal	Abnormal
	Normal	83	2 ii	Normal	131	2 iv	Normal	142	0
	Abnormal	0	58	Abnormal	0	10	Abnormal	0	1
		***EBF1***			***PAX5***			**PAR1**	
		Normal	Abnormal		Normal	Abnormal		Normal	Abnormal
	Normal	133	0	Normal	95	2 iv	Normal	138	2 i
	Abnormal	1 v	9	Abnormal	2 v	43, 1 iii	Abnormal	0	3

This shows the nature of the concordant and discordant calls for each of the eight genes and the PAR1 region.

Disagreement categories are shown as roman numerals. i = no SNP array probes covering the abnormality–

meaning that due to the array design it is impossible to make a call. ii = MLPA result was informed by a single

MLPA probe. iii = The CBS algorithm failed to pick up a small focal abnormality, which could be called visually

by inspection of the Log R Ratio (LRR) data such that they would be concordant with the MLPA calls. iv = The

two techniques disagree despite sufficient probe coverage. v = SNP6.0 detected an abnormality which could not

be detected by MLPA due to lack of probe coverage.

**Table 2:** Full table of results broken down by patient ID including the nature of *IKZF1* discordance.

Gene/Region	Patient ID	CNA-MLPA	CNA-SNP6.0: CBS	CNA-SNP6.0: manual	Discordance type	UKALL-CNA: MLPA	UKALL-CNA: SNP-CBS	UKALL-CNA: SNP-manual
*IKZF1*	19732	deletion ex4-7	normal	deletion ex4-7	iii	IR/PR	IR/PR	IR/PR
*IKZF1*	20035	deletion ex2-7	normal	deletion ex2-7	iii	IR/PR	GR	IR/PR
*IKZF1*	20753	deletion ex4-8	normal	deletion ex4-8	iii	IR/PR	IR/PR	IR/PR
*IKZF1*	22964	deletion ex4-7	normal	deletion ex4-7	iii	IR/PR	IR/PR	IR/PR
*IKZF1*	22388	deletion ex4-7	normal	deletion ex4-8	iii	IR/PR	GR	IR/PR
*IKZF1*	10054	deletion ex4-7	normal	deletion ex4-7	iii	IR/PR	GR	IR/PR
*IKZF1*	10062	deletion ex4-7	normal	deletion ex4-8	iii	IR/PR	IR/PR	IR/PR
*IKZF1*	11560	deletion ex4-7	normal	—	iv	IR/PR	GR	GR
*IKZF1*	11741	deletion ex4-7	normal	deletion ex4-7	iii	IR/PR	GR	IR/PR
*ETV6*	8947	deletion	normal	—	iv	IR/PR	IR/PR	IR/PR
*CDKN2A*	20753	deletion	normal	—	i	IR/PR	IR/PR	IR/PR
*CDKN2A*	22572	deletion	normal	—	i	IR/PR	GR	GR
*CDKN2A*	22689	deletion	normal	—	i	IR/PR	IR/PR	IR/PR
*CDKN2B*	21324	deletion	normal	—	ii	IR/PR	IR/PR	IR/PR
*CDKN2B*	22572	deletion	normal	—	ii	IR/PR	GR	GR
*RB1*	10958	deletion	normal	—	iv	IR/PR	GR	GR
*RB1*	20874	gain	normal	—	iv	IR/PR	IR/PR	IR/PR
*EBF1*	3278	normal	loss	—	v	IR/PR	IR/PR	IR/PR
*PAX5*	9262	gain	normal	—	iv	IR/PR	IR/PR	IR/PR
*PAX5*	10077	gain	normal	gain	iii	GR	GR	GR
*PAX5*	10442	gain	normal†	—	iv	IR/PR	IR/PR	IR/PR
*PAX5*	20515	normal	loss	—	v	GR	GR	GR
*PAX5*	11957	normal	loss	—	v	GR	GR	GR
PAR1	11403	rearrangement	normal	—	i	IR/PR	GR	GR
PAR1	21819	rearrangement	normal	—	i	IR/PR	IR/PR	IR/PR

Most *IKZF1* cases have an exon 4 to 7 loss, these are much harder for the circular binary segmentation (CBS) algorithm to detect because of the occurrence of them after a gap in coverage of a covered region. The UKALL-CNA-SNP6.0 CBS column gives the calls from the automated CBS algorithm in DNACopy. The entries in the UKALL-CNA-SNP6.0 manual column give the result from manual calling of events using the Log *R* Ratio (LRR) plots, only cases where the call changes to that of the UKALL-CBS-SNP6.0 column are shown, in cases where the call remained the same - is recorded. Only one of the cases had a spanning deletion of exons 2–7. The classification of the nature of dis/concordance is as in Table 1. i = no SNP array probes covering the abnormality - meaning that due to the array design it is impossible to make a call. ii = MLPA result was informed by a single MLPA probe. iii = The CBS algorithm failed to pick up a small focal abnormality, which could be called visually by inspection of the LRR data such that they would be concordant with the MLPA calls. iv = The two techniques disagree despite sufficient probe coverage. In one of these six cases of disagreement FISH data was available and validated the normal calls made by SNP6.0 this is indicated by †. v = SNP6.0 detected an abnormality which could not be detected by MLPA due to lack of probe coverage.

### MLPA and SNP6.0 probe locations

**Figure 2:**
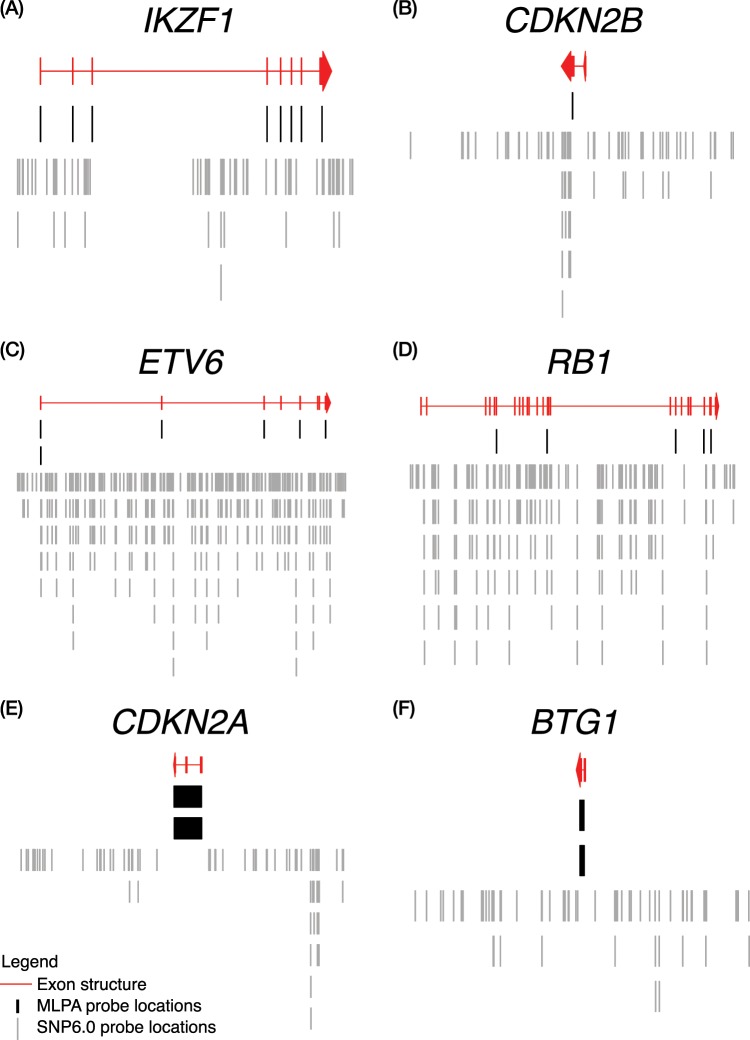
The location of Individual SNP6.0 and MLPA probes. The location of Individual SNP6.0 probes as points of Log *R* Ratio (LRR) are shown in grey verses the MLPA probes as black lines/rectangles for the genes (**A**) *IKZF1* (**B**) *CDKN2B* (**C**) *ETV6* (**D**) *RB1* (**E**) *CDKN2A* (**F**) *BTG1*. Exons from genes are plotted as boxes in red. MLPA probe sequences were obtained from MRC-Holland and aligned to hg19 using BLAT. Where the MLPA probe aligned to more than one region of the genome the top scoring BLAT alignment (without gaps) was then chosen as the definitive location for each MLPA probe.

**Figure 3:**
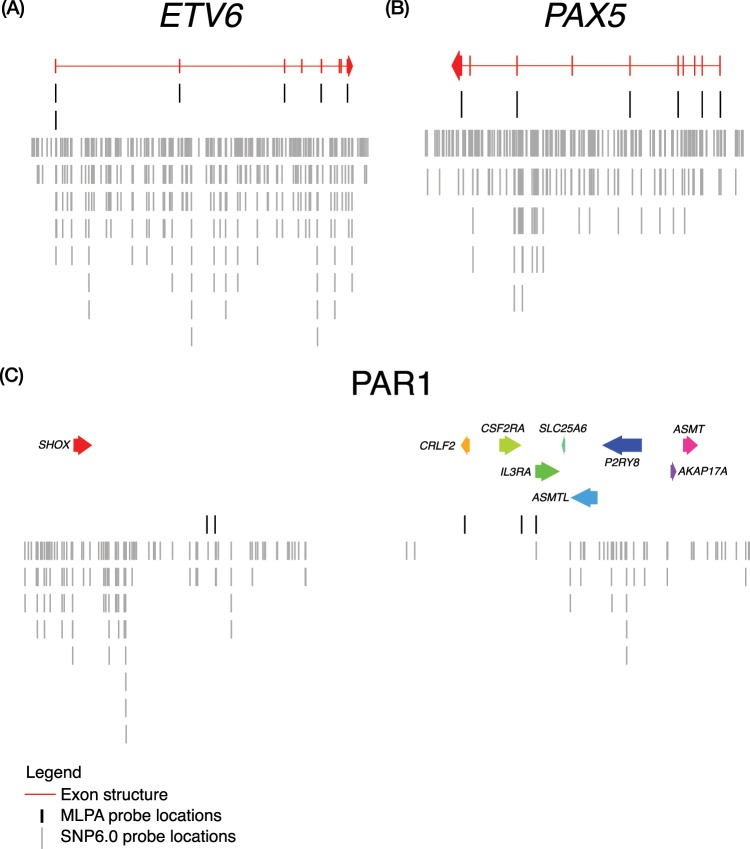
The location of Individual SNP6.0 and MLPA probes. The location of Individual SNP6.0 probes as points of Log *R* Ratio (LRR) are shown in grey verses the MLPA probes as black lines for the genes (**A**) *EBF1* (**B**) *PAX5* (**C**) PAR1 region. Exons from genes are plotted as boxes in red. In (**C**) the coloured arrows represent individual genes. MLPA probe sequences were obtained from MRC-Holland and aligned to hg19 using BLAT, the highest scoring alignment with zero gaps was then chosen as the location of each MLPA probe.

**Figure 4:**
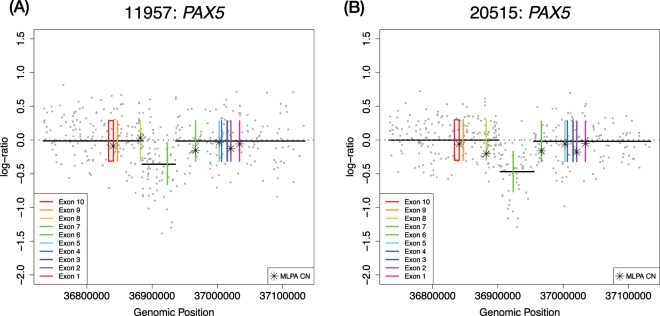
*PAX5* losses revealed by SNP6.0 in cases missed by MLPA. Patient ID and gene name are shown in the title of each plot, individual SNP6.0 data points of Log *R* Ratio (LRR) are shown in grey, copy number calls by the binary segmentation algorithm are shown as black lines. The copy normal LRR value of zero is shown as a grey dotted line, exons from genes are plotted as plotted boxes in rainbow colour, and MLPA data points converted into the LRR scale are shown as stars. (**A,B**) both show examples of a missed loss events in *PAX5* exon 7 which could not be called in MLPA owing to a lack of coverage.

For the genes represented in the P335 MLPA kit, we investigated the precise location of MLPA probes in comparison to the SNP6.0 probes in order to determine whether their positioning may explain any of the observed differences in calls. The top scoring BLAT^21^ alignment for each probe uniquely located each probe sequence (provided by MRC Holland, Amsterdam, The Netherlands) to a single fully aligned region of the genome without gaps. The co-ordinates of the aligned MLPA probe locations are given in Supplementary Table S3 and plotted in Figs. 2 and 3. Most of the genes targeted by the MLPA kit were well covered by probes on the SNP6.0 array. However, a few genes had poor probe coverage on the SNP6.0 array: *CDKN2A* lacked probes internal to the gene; while the genes *CRLF2*/*CSF2RA*/*IL3RA* within the PAR1 region were covered by a single probe. The MLPA kit had been designed specifically to detect copy number events within these genes, hence the probes were well positioned within the exons. However, not every exon of every gene was covered, as is the case in *RB1*, *EBF1* and *PAX5*. The partial coverage of *EBF1* and *PAX5* resulted in MLPA missing three small deletions, which were detected by SNP6.0. These three cases are illustrated in Fig. 1(B) (*EBF1*) and Fig. 4 (*PAX5*). *CDKN2B* was also only covered by a single MLPA probe covering exon 1. Issues arising from these differences in coverage are discussed in the next section.

### Examination of discordant calls

Of the 25 discordant calls made by the SNP6.0 pipeline using the CBS algorithm, subsequent manual examination of the LRR data plots produced by DNACopy for these calls revealed that nine of the abnormalities were visible by eye but had evaded detection by the CBS algorithm (disagreement category iii). Manual inspection of LRR plots in these nine cases allowed for concordant deletion of these otherwise missed abnormalities, eight in *IKZF1* and one in *PAX5*. For the *IKZF1* gene, 21/29 deletions were called, with eight deletions missed by the CBS algorithm but visible in the LRR data plots, in seven out of these eight cases, *IKZF1* deletions spanned exons 4 to 7/8. In these cases, the CBS algorithm failed to call focal abnormalities which flanked a region of the *IKZF1* gene, where there was no probe coverage within intron 3 (between exons 3 and 4, ~42Kb in length) on the SNP6.0 platform. It should be noted that all array platforms lack coverage of this region of *IKZF1*, due to the repetitive nature of the reference genome sequence of this region and the inability of array platforms to target probes complementary to this region in a sequence-specific way. In these cases, visual inspection allowed for manual calling of the abnormality; an example of an event downstream of the “*IKZF1* hole” missed by the CBS algorithm is shown in Fig. 1(C). Here the black line indicates that segmentation is normal, but LRR data points reveal a deletion concordant with MLPA data. A further two cases of exon 4–5 and exon 4–8 deletions were successfully detected by CBS, so whilst it was possible to detect these deletions, the presence of a gap in coverage spanning exons 3–4 clearly presents problem for the CBS algorithm when dealing with events immediately upstream of the gap. This issue was easily surmounted by manually inspecting plotted LRR data for the *IKZF1* gene.

A single gain within *PAX5* was missed by the CBS algorithm. It was highly focal and evidenced by a single probe, which showed a clearly distinct LRR value of ~1.5. Given the gain was only identified by one probe, it would be impossible and unwise for an automated method to call. We employed a minimum number of markers cut-off of five for the CBS algorithm in our study, meaning that for an event to be detected at least five probes must be involved with a statistically significant deviation. We also tested using a minimum cut-off of four, three and two markers; none of these options resulted in any of the nine missed events being called. These missed events were not due to the CNA thresholds that we applied over the top of the segmentation data, used to enable categorical labelling of gains, amplifications, losses, and deletions (as illustrated in Supplementary Fig. S3). Simply, these missed calls occurred because the CBS algorithm did not segment the events.

In the remaining 16 discordant calls, we found that eight calls resulted from design differences in the two platforms, such that abnormalities detected in one could not be observed in the other owing to a lack of probe coverage; these cases belonged to disagreement categories (i) SNP6.0 lacking probes, and (v) MLPA lacking probes. For category (i) Fig. 1(B) shows an example of a focal deletion within *CDKN2A*, which was not detectable by SNP6.0 because no probes lie within the gene itself (Fig. 2(E)); this situation occurred in three cases. We also observed this type of discordance in two cases involving the PAR1 region, where the abnormalities occurred in a region within *CSF2RA*/*IL3RA*, as there are no probes on the array in this area (Fig. 3(C)). Importantly, none of these cases (*n* = 8) reflected a failure of the CBS algorithm to detect the events, they were simply not observable using this particular array. The MLPA kit also failed to detect events due to the absence of probes in the appropriate region (discordance category v); we observed three cases of this type. The SNP array was able to detect a focal loss in *EBF1* (Fig. 1(B)). There were also two cases of focal deletions in *PAX5* observed on the SNP array that were not detected by the MLPA kit. These both occurred in exon 7 of *PAX5*, which has no coverage by MLPA probes (Fig. 3(B)).

**Figure 5:**
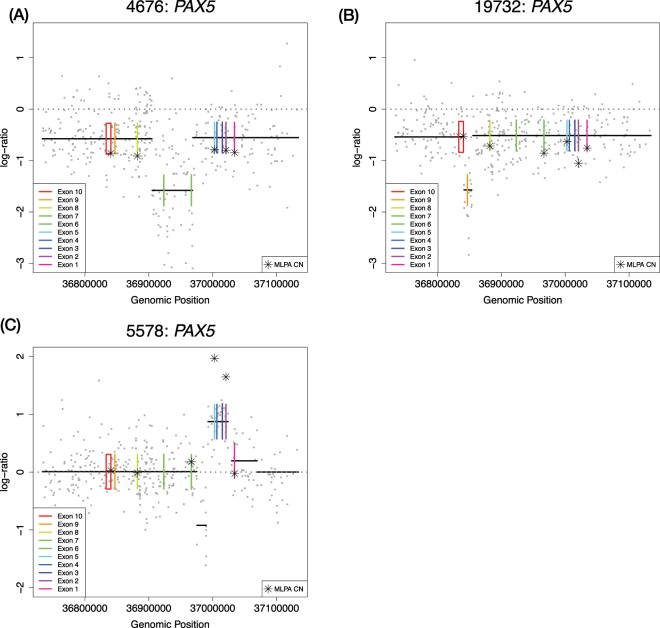
Extra information revealed by SNP6.0 in cases where calls were overall MLPA and SNP6.0 were concordant. Patient ID and gene name are shown in the title of each plot, individual SNP6.0 data points of Log *R* Ratio (LRR) are shown in grey, copy number calls by the binary segmentation algorithm are shown as black lines. The copy normal LRR value of zero is shown as a grey dotted line, exons from genes are plotted as plotted boxes in rainbow colour, and MLPA data points converted into the LRR scale are shown as stars. (**A**) Shows a concordant *PAX5* call in this case both techniques reported a deletion, however, the SNP6.0 CBS segmentation show a nested deletion event in exons 6–7 which was not detectable using the MLPA kit because these exons have no coverage (Fig. 3(B)). (**B**) Similarly shows a nested deletion of exon 9 in *PAX5*. (**C**) Here both techniques show a gain, the gain here taking precedence in SNP6.0 as the reported event as it is higher than the lowest nested deletion event, (CBS copy number values of 3.6 vs 1.1 respectively). The nested loss here was not detectable via MLPA as no probe covers this region, additionally this copy number pattern is characteristic of a *PAX5* rearrangement which is of diagnostic significance.

It is also worth highlighting that even when abnormalities called by both methods were concordant, the SNP6.0 pipeline was able to reveal extra details and events not observable by MLPA. Figure 5 highlights three cases of concordant *PAX5* calls, which revealed extra levels of detail regarding copy number events by the SNP6.0 array using our pipeline that were not observable using the MLPA kit.

There were two cases where MLPA detected a focal deletion of *CDKN2B* with just a single probe (type ii). Both of these discordant cases occurred within *CDKN2B*, in which normal values of copy were observed from the many SNP6.0 probes in this region. Here we consider the many repeated observations from the array to be the most reliable compared to the single observation from the MLPA kit; an example is given in Fig. 1(F), and relative probe locations are shown in Fig. 2(B). These two cases illustrate the potential limitation in measuring the copy number of a gene using a single probe in the MLPA kit, which is error prone and unreliable (in part due to the inability of MLPA probes to tolerate SNPs) versus the multiple observations taken from the SNP6.0 array. For this reason, normally ≥2 adjacent MLPA probes are required for calling a copy number alteration^19^.

We observed only six calls in total where the two techniques were in disagreement and the supporting evidence was not concordant despite sufficient probe coverage, (disagreement category iii). In all these cases, MLPA detected an abnormality but there was no obvious or robust deflection of LLR data points by SNP array. In one of these cases, FISH data was available for the gene in question (*PAX5*), which further corroborated the SNP6.0 call (highlighted in Table 2). In the remaining discordant cases, it was possible that differences in sensitivity of the two techniques to sub-clonal events means that events detected by one are not observable by the other. We also checked whether any patient samples were repeatedly discordant indicating a sample swap. All discordant cases, came from different samples, with the exception of a single *CDKN2A* and *CDKN2B* deletion which occurred within the same patient (22572). As these two genes are adjacently located, it was likely the same event detected by MLPA.

### Effect of using SNP6.0 array data with the UKALL-CNA classifier

Previously, we designed^4^ and validated^22^ a copy number based ALL risk classifier (UKALL-CNA classifier), which segregates patients based on the copy number states of the eight genes and the PAR1 region included in the MLPA P335 kit. This classifier assigns patients into three groups: good risk (GR), intermediate risk (IR), and poor risk (PR), with significantly different outcomes. In this study, we have grouped together the IR and PR group as the major prognostic findings from our discovery and validation studies was that B-other-ALL patients with a GR CNA profile had an excellent outcome^4^ and B-other-ALL formed the bulk of this cohort (121/143 cases). We used the same algorithm to segregate 143 samples by UKALL-CNA classifier using both the MLPA CNA calls and our SNP6.0 pipeline with the fully automated CBS or manually augmented calls. Using manually augmented calling, 97% (*n* = 139) of patients would have been assigned to the same CNA-risk profile (Table 3). Here four patients with different profiles, were assigned to GR using the SNP calls in place of an IR/PR assignment using the original MLPA calls. Using the fully automated SNP CBS calls an additional four cases, eight in total were moved from the IR/PR group to GR (Table 3). Table 2 shows the changes in UKALL-CNA classifier result for each discordant case.

**Table 3:** Changes in UKALL-CNA classification due to changes in CNA calls between the two techniques.

		Classification using SNP manual calling		Classification using SNP automated CBS	
		GR	IR/PR	GR	IR/PR
CNA risk MLPA	GR	64	0	64	0
	IR/PR	4	75	8	71

Showing both manual calling of otherwise discordant calls in *IKZF1* and *PAX5* (left) and calling with CBS algorithm alone (right). Individual changes in the UKALL-CNA classifier outcome are listed for each patient in Table 2.

## Discussion

In this study, we have validated the use of SNP arrays to call MLPA defined UKALL-CNA profiles, which have been previously validated across several patient cohorts^22^. We investigated concordance between two different techniques: SNP6.0 array and MLPA for the detection of copy number alterations across key genes somatically altered in B-ALL. Our aim was to investigate how well SNP arrays perform for risk classification of B-other-ALL using the UKALL-CNA classifier^4,22^ in comparison to the MLPA kit^19^. These results are important because they provide good evidence that the UKALL-CNA profile can be reliably called using a different platform. Hence, future protocols, which decide to adopt the UKALL-CNA classifier into their risk stratification algorithm, can be confident that diagnostic laboratories using SNP arrays will be calling the same profile identified by the research studies using MLPA. Although we have focussed on validating our own CNA profile using a single MLPA kit (P335), this research has implications for other MLPA derived profiles; for example the *IKZF1*^plus^ profiles recently defined and validated by the BFM and AEIOP study groups^7^. SNP arrays are increasingly being used in a diagnostic setting to detect a wide range of losses, gains, and loss of heterozygosity in cancer. This is evidenced by the fact that the American College of Medical Genetics and Genomics (ACMG) and the Cancer Genomics Consortium (CGC) have issued guidelines for diagnostic laboratories analysing SNP arrays^23^. Moreover, chromosomal ploidy (for example, near-haploidy and low hypodiploidy) and arm-level events normally derived from conventional karyotyping are also detectable using SNP arrays which are key in ALL^24^. As SNP arrays are genome-wide, their routine application means that new risk associated CNA events in other genes can be immediately investigated. Additionally, our SNP6.0 pipeline employs automated copy number calling, using the CBS algorithm^16^. Automated calling via the CBS algorithm has advantages over manual calling, being much quicker, and scaling systematically beyond a handful of genes to the whole-genome in an unbiased way, while returning only statistically significant copy number events^17^. Our pipeline uses the popular open source packages PennCNV^13,14^ and DNACopy^16,17^ and can be easily replicated elsewhere, additionally, data exported from either Affymetrix Genotyping Console / Chromosome Analysis Suite and Illumina Genome Studio can be analysed with these tools to yield high quality segmentation via the CBS algorithm. The freely available Nexus Copy Number Plugin for Genome Studio also has an implementation of the CBS algorithm, which is usable with Illumina SNP array data. The CBS algorithm is also available for CNA calling of high-throughput sequencing data in methods such as CNVKit^25^.

Using our SNP6.0 analysis pipeline, we found 98% concordance between SNP6.0 and MLPA across 1,287 individual calls and 99% concordance if we allowed for manual inspection of *IKZF1* and *PAX5* LRR plots. Of these 16 remaining discordant calls, only five cases (4%) were undetectable by SNP6.0 owing to design limitations in the location of probes.

The UKALL-CNA risk classifier validated in this study is a useful tool to help segregate patients with differential relapse risk for treatment stratification. Here we have assigned UKALL-CNA profile based risk classification using both manual and automated SNP copy number calls for each patient in comparison to the original MLPA based classification. We observed a small proportion of patients who would have been assigned to different risk groups (3% for manual and 6% for automated calls). Most of the inconsistencies (four out of eight) arose from the lack of information due to automatically calling the copy number states of *IKZF1*. Among those cases where a lack of coverage of probes on the array led to three and two events being missed in *CDKN2A* and PAR1 respectively, (disagreement category i in Tables 1 and 2), the resultant differences in UKALL-CNA profile risk classification over MLPA informed classification would potentially lead to two patients (included in the four above) being assigned to a different CNA risk group.

Our observations have indicated that the SNP6.0 pipeline, employing the CBS algorithm and manually augmented calling for *IKZF1*, could be used as an alternative to MLPA based copy number detection systems in ALL CNA profile based risk classification. It has also highlighted the need to improve CBS based callers in order to cope with missing information, such as the “*IKZF1* hole” in the SNP6.0 and other array platforms, which is also problematic for genomic alignments of high-throughput sequencing data. Finally, we highlight that other array-based platforms will require similar scrutiny with respect to probe location and coverage before being employed to call profiles derived using targeted copy number analysis techniques like MLPA.

## Supplementary information

Supplementary Information.
