# Supplementary material for: Concordance of copy number abnormality detection using SNP arrays and Multiplex Ligation-dependent Probe Amplification (MLPA) in acute lymphoblastic leukaemia
Source: Sci Rep. 2020 Jan 8;10:45. doi: 10.1038/s41598-019-56972-0 (PMC6949215; doi:10.1038/s41598-019-56972-0)
Supplement: Supplementary file 1 — Supplementary Information. [file 41598_2019_56972_MOESM1_ESM.pdf]

# Concordance of copy number abnormality detection using SNP arrays and Multiplex Ligation-dependent Probe Amplification (MLPA) in acute lymphoblastic leukaemia

Authors: Matthew Bashton[1], Robin Hollis[1], Sarra Ryan[1], Claire Schwab[1], John Moppett[2], Christine J Harrison[1], Anthony V Moorman[1], Amir Enshaei[1]*

1. Translational and Clinical Research Institute, Faculty of Medical Science, Centre for Cancer, Newcastle University, Newcastle upon Tyne, UK
2. Department of Haematology, Royal Hospital for Sick Children, Bristol, UK

* Corresponding author

E-mail: amir.enshaei@newcastle.ac.uk

# Supplemental methods

## Representativeness of cohort

To check the representativeness of the analysed cohort used we tested clinical variables, including survival data, to make sure the analysed cohort is representative of the two trials. Details on the composition of our cohort and the results of statistical testing are given in Tables S1 and S2 which show our analysed cohort to be representative.

## Circular binary segmentation (CBS) based automated copy number abnormality calling

We employed the CBS algorithm over hidden Markov model (HMM) based callers (such as PennCNV's caller) as the CBS algorithm is more appropriate for somatic copy number calling as it reports copy as a continuous variable. This is better able to cope with the nature of CNAs in somatic cancer samples as the underlying copy values are often, due to a mix of sub-clonal populations and/or often have normal tissue contamination. HMMs which operate over B-allele frequency data are flawed for somatic CNA calling as the HMMs only have a finite number of states which reflect whole integer values of copy. Whilst appropriate for germline calling these states will lead to intermediate values of copy being coerced into the nearest whole integer value, this tends to flatten out events and mask gene rearrangements and Chromothripsis in somatic samples. We employed the default DNACopy recommended parameters for segmentation, with the exception of raising the minimum number of probes required for calling an event to five from two, as this eliminated the risk of unwanted hyper-segmentation. Called copy number segments from the CBS algorithm were then collated and visualised for all genes of interest using functions implemented in R. These functions utilised the Gviz[1] and DNACopy packages in Bioconductor to generate regional plots of genes on interest.

For the automated calling, all statistically significant segments were called regardless of copy number value using DNACopy. We then applied thresholds over the top of the called segments. An actual copy number value of ≤ 1.7 was employed to indicate a loss within a gene, with deletions reported when values of copy < 1 were observed, *i.e*. less than one chromosomes' worth of material remaining. Gains were reported with values ≥ 2.3 and amplifications with values > 3 *i.e*. more than one extra chromosome of material. This means that regions of number copy > 1.7 and < 2.3 were considered normal, with 2.0 representing an exactly diploid value. These thresholds for normal copy were chosen as the median value of called copy for all called segments in 143 arrays was 1.98, with a narrow standard deviation of 0.51. The kernel density plot of these values Supplementary Fig. S2 shows that the vast majority of our calls lie safely within the bounds of < 2.3 and > 1.7 allowing for events to be robustly called. Supplementary Fig. S3 shows idealised simulated calls for normal, gain, amplification, loss and deletion with our chosen thresholds indicated as Log *R* ratio values.

In cases with more than one event within a gene, the highest deviation from a normal copy of 2 was reported *e.g*. if a gene had a gain, at a value of 2.4 but also a deletion, at a value of 0.8, then the deletion event with delta of 1.2 as opposed to 0.4 for the gain, from a normal copy of 2, would take precedence as the "event" for that gene. The pseudoautosomal region 1 (PAR1) was handled differently, here we are looking for copy number aberrations, which indicate a rearrangement of *CRLF2* in reference to the base copy number value of *SHOX*. We may have a diploid reference copy value for *SHOX* (normal for males and females) and a deletion occurring amongst the *CRLF2*/*CSF2RA*/*IL3RA* genes leading to a copy number of one. Or, alternatively, three copies of the X chromosome (trisomy), but with a deletion event leading to a copy number of two (diploid) amongst *CRLF2*/*CSF2RA*/*IL3RA*. Both of these

situations could lead to a localised deletion, potentially producing a *P2YR8-CRLF2* fusion, such deletions are important because *CRLF2* rearrangements, and/or deregulation is involved in lymphoid transformation of B-ALL[2,3] and is associated with poor outcome[4].

## MLPA probe locations

Full probe sequences for the SALSA MLPA kit P335 were provided by MRC Holland, Amsterdam, The Netherlands, and aligned to hg19 using The BLAST-like Alignment Tool (BLAT)[5] (Kent Informatics, Inc., Santa Cruz, California). Co-ordinates for the top matching hit with zero gapped alignment were then used for each MLPA probe location.

## Table S1: ALL97

| | All B-other patients | Not analysed B-other patients | Analysed B-other patients | |
|---|---|---|---|---|
| | 505 | 483 | 22 | ***p*-value*** |
| **Sex** | | | | 0.857 |
| F | 239(47) | 229(47) | 10(45) | |
| M | 266(53) | 254(53) | 12(55) | |
| **Age** | | | | 0.001 |
| 0-9 | 356(71) | 348(72) | 8(36) | |
| 10-16 | 125(25) | 114(24) | 11(50) | |
| 16+ | 23(5) | 20(4) | 3(14) | |
| **WCC** | | | | 0.772 |
| 0-49 | 398(79) | 382(79) | 16(73) | |
| 50-99 | 55(11) | 52(11) | 3(14) | |
| 100+ | 52(10) | 49(10) | 3(14) | |
| **NCI risk** | | | | 0.002 |
| SR | 275(54) | 270(56) | 5(23) | |
| HR | 230(46) | 213(44) | 17(77) | |
| **CR** | | | | 0.57 |
| Yes | 498(99) | 476(99) | 22(100) | |
| No | 7(1) | 7(1) | 0(0) | |
| **Death** | | | | 0.507 |
| Yes | 109(22) | 103(21) | 6(27) | |
| No | 396(78) | 380(79) | 16(73) | |
| **Relapse** | | | | 0.169 |
| Yes | 120(24) | 112(24) | 8(36) | |
| No | 378(76) | 364(76) | 14(64) | |
| **Event** | | | | 0.512 |
| Yes | 152(30) | 144(30) | 8(36) | |
| No | 353(70) | 339(70) | 14(64) | |
| | | | | ***p*-value†** |
| **EFS @ 5 years** | 73%(69-77) | 74%(69-77) | 68%(45-83) | 0.53 |
| **RR @ 5 years** | 22%(18-26) | 21%(18-25) | 32%(17-55) | 0.18 |
| **OS @ 5 years** | 83%(79-86) | 83%(79-86) | 82%(58-93) | 0.54 |

* Fisher's exact test

† Log-rank test

**Table S1. Demographic, clinical, genetic and outcome features of patients with B-other acute lymphoblastic leukaemia (ALL) according to whether or not pre-treatment samples were analysed and not analysed by SNP for ALL97 trial**. Table shows a descriptive analysis of the clinically relevant and outcome variables for: all B-other patients in the trial,

B-other patients who were analysed in this study, and those not analysed patients (not all had diagnosis SNP6.0 arrays). To make sure the tested cohort is not significantly different to the untested tested portion of the trial in terms of clinically relevant variables, Fisher's exact test was used. In addition, to make sure there is no significant differences between the tested and untested portion of the cohort, regarding clinical outcome (EFS, RR and OS), we have used the log-rank test. The results from this analysis shows that there are no significant differences in terms of clinically relevant variables and outcome for the B-other cases that were included and not included in this study, demonstrating our analysed cohort is representative of the whole ALL97 trial. With the exception of age and national cancer institute (NCI) risk score, both of these are likely due to the small cohort size of 22 we analysed from this trial, regardless, since survival and other clinical variables are not significantly different this would have not resulted in an unrepresentative cohort. WCC: white cell count; NCI: national cancer institute (SR: WCC<50 and Age<10); MRD: minimal residual disease; CR: complete remission; EFS: event free survival; RR: relapse rate; OS: overall survival.

## Table S2: UKALL2003

| | All B-other patients | Not analysed B-other patients | Analysed B-other patients | |
|---|---|---|---|---|
| | 780 | 659 | 121 | ***p*-value*** |
| **Sex** | | | | 0.824 |
| F | 323(41) | 274(42) | 49(40) | |
| M | 457(59) | 385(58) | 72(60) | |
| **Age** | | | | 0.2 |
| 0-9 | 489(63) | 406(62) | 83(69) | |
| 10-16 | 191(24) | 163(25) | 28(23) | |
| 16+ | 100(13) | 90(14) | 10(8) | |
| **WCC** | | | | 0.158 |
| 0-49 | 628(81) | 536(81) | 92(76) | |
| 50-99 | 84(11) | 71(11) | 13(11) | |
| 100+ | 68(9) | 52(8) | 16(13) | |
| **NCI risk** | | | | 0.946 |
| SR | 389(50) | 329(50) | 60(50) | |
| HR | 391(50) | 330(50) | 61(50) | |
| **MRD** | | | | 0.053 |
| 0% | 141(18) | 127(19) | 14(12) | |
| 0-0.005% | 140(18) | 119(18) | 21(17) | |
| 0.005-0.01% | 47(6) | 36(5) | 11(9) | |
| 0.01-0.1% | 159(20) | 130(20) | 29(24) | |
| 0.1-1.0% | 106(14) | 84(13) | 22(18) | |
| 1-5% | 36(5) | 32(5) | 4(3) | |
| >5% | 35(4) | 25(4) | 10(8) | |
| NA | 116(15) | 106(16) | 10(8) | |
| **CR** | | | | 0.383 |
| Yes | 766(98) | 646(98) | 120(99) | |
| No | 14(2) | 13(2) | 1(1) | |
| **Death** | | | | 0.968 |
| Yes | 102(13) | 86(13) | 16(13) | |
| No | 676(87) | 571(87) | 105(87) | |
| **Relapse** | | | | 0.354 |
| Yes | 95(12) | 77(12) | 18(15) | |
| No | 669(88) | 567(88) | 102(85) | |
| **Event** | | | | 0.827 |
| Yes | 147(19) | 125(19) | 22(18) | |
| No | 631(81) | 532(81) | 99(82) | |
| | | | | ***p*-value†** |
| **EFS @ 5 years** | 82%(80-85) | 82%(79-85) | 82%(74-88) | 0.73 |
| **RR @ 5 years** | 12%(10-15) | 11%(9-14) | 15%(10-23) | 0.43 |
| **OS @ 5 years** | 89%(86-91) | 89%(86-91) | 90%(83-94) | 0.84 |

* Fisher's exact test

† Log-rank test

**Table S2. Demographic, clinical, genetic and outcome features of patients with B-other acute lymphoblastic leukaemia (ALL) according to whether or not pre-treatment samples were analysed and not analysed in the study for UKALL2003 trials**. Table shows a descriptive analysis of the clinically relevant and outcome variables for: all B-other patients in the trial, B-other patients who were analysed in this study, and those not analysed patients (not all had diagnosis SNP6.0 arrays). To make sure the tested cohort is not significantly different to the untested tested portion of the trial in terms of clinically relevant variables, Fisher's exact test was used. In addition, to make sure there is no significant differences between the tested and untested portion of the cohort, regarding clinical outcome (EFS, RR and OS), we have used the log-rank test. The results from this analysis shows that there are no significant differences in terms of clinically relevant variables and outcome for the B-other cases that were included and not included in this study, demonstrating our analysed cohort is representative of the whole UKALL2003 trial. WCC: White cell count; NCI: national cancer institute (SR: WCC<50 and Age<10); MRD: minimal residual disease; CR: complete remission; EFS: event free survival; RR: relapse rate; OS: overall survival.

| Gene, Exon targeted<br>Or Region, Gene | Chromosome | Start (bp) | End (bp) |
|---|---|---|---|
| *IKZF1*, exon 1 | 7 | 50,344,475 | 50,344,532 |
| *IKZF1*, exon 2 | 7 | 50,358,663 | 50,358,736 |
| *IKZF1*, exon 3 | 7 | 50,367,249 | 50,367,307 |
| *IKZF1*, exon 4 | 7 | 50,444,301 | 50,444,360 |
| *IKZF1*, exon 5 | 7 | 50,450,254 | 50,450,309 |
| *IKZF1*, exon 6 | 7 | 50,455,010 | 50,455,082 |
| *IKZF1*, exon 7 | 7 | 50,459,458 | 50,459,525 |
| *IKZF1*, exon 8 | 7 | 50,468,525 | 50,468,588 |
| *ETV6*, exon 1a | 12 | 11,802,943 | 11,803,004 |
| *ETV6*, exon 1b | 12 | 11,803,060 | 11,803,127 |
| *ETV6*, exon 2 | 12 | 11,905,436 | 11,905,494 |
| *ETV6*, exon 3 | 12 | 11,992,072 | 11,992,131 |
| *ETV6*, exon 5 | 12 | 12,022,383 | 12,022,444 |
| *ETV6*, exon 8 | 12 | 12,044,099 | 12,044,163 |
| *CDKN2A*, exon 4 | 9 | 21,967,821 | 21,967,894 |
| *CDKN2A*, exon 2 | 9 | 21,974,957 | 21,975,028 |
| *CDKN2B*, exon 2 | 9 | 22,005,812 | 22,005,893 |
| *RB1*, exon 6 | 13 | 48,923,212 | 48,923,282 |
| *RB1*, exon 14 | 13 | 48,953,430 | 48,953,499 |
| *RB1*, exon 19 | 13 | 49,030,372 | 49,030,441 |
| *RB1*, exon 24 | 13 | 49,047,204 | 49,047,270 |
| *RB1*, exon 26 | 13 | 49,051,487 | 49,051,563 |
| *BTG1*, exon 2 | 12 | 92,537,568 | 92,537,641 |
| *BTG1*, exon 1 | 12 | 92,539,154 | 92,539,219 |
| *EBF1*, exon 1 | 5 | 158,526,608 | 158,526,681 |
| *EBF1*, exon 10 | 5 | 158,204,438 | 158,204,505 |
| *EBF1*, exon 14 | 5 | 158,139,231 | 158,139,292 |
| *EBF1*, exon 16 | 5 | 158,125,249 | 158,125,316 |
| *PAX5*, exon1 | 9 | 37,034,271 | 37,034,332 |
| *PAX5*, exon 2 | 9 | 37,020,668 | 37,020,735 |
| *PAX5*, exon 5 | 9 | 37,002,698 | 37,002,763 |
| *PAX5*, exon 6 | 9 | 36,966,621 | 36,966,679 |
| *PAX5*, exon 8 | 9 | 36,882,065 | 36,882,123 |
| *PAX5*, exon 10 | 9 | 36,840,474 | 36,840,535 |
| PAR1, *SHOX*, area 1 | X | 835,205 | 835,205 |
| PAR1, *SHOX* ,area 2 | X | 850,580 | 850,580 |
| PAR1, *CRLF2*, exon 4 | X | 1,321,273 | 1,321,273 |
| PAR1, *CRLF2A*, exon 16 | X | 1,428,385 | 1,428,385 |
| PAR1, *IL3RA*, exon 1 | X | 1,455,700 | 1,455,770 |

**Table S3**. MLPA probe locations for the SALSA MLPA kit P335 in the hg19 human genome. MLPA sequences were obtained from MRC Holland, Amsterdam, The Netherlands, and aligned to hg19 using The BLAST-like Alignment Tool (BLAT) [5] (Kent Informatics, Inc., Santa Cruz, California). Co-ordinates for the top matching hit with zero gapped alignment were then used for each MLPA probe location. Note exon numbering is as per P335 kit documentation.

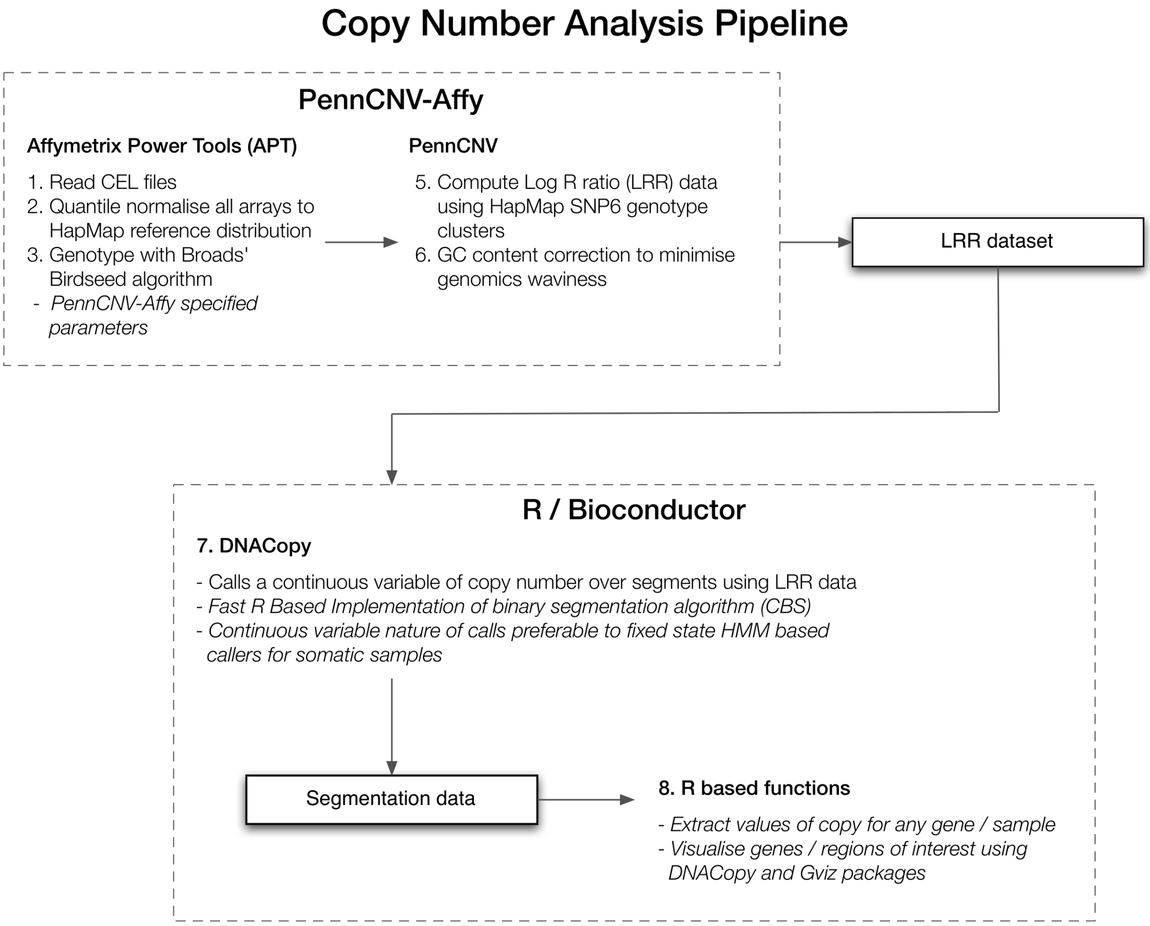


**Fig. S1. Overview of Copy Number Analysis Pipeline used to generate calls from SNP6.0 array data.**

The PennCNV-Affy pipeline itself utilises the Affymetrix Power Tools Linux command-line set of programs for initial data extraction and normalisation. Log *R* ratio (LRR) calculation is performed by PennCNV[6] using reference genotype clusters from the HapMap 3 project which were also generated using SNP6.0 arrays. Genomic waviness GC-content correction for hg19 is then performed using PennCNV[7]. The DNACopy[8] package in Bioconductor[9] is then used to perform copy number segmentation using the CBS algorithm[10], finally custom R [11] based functions are used to visualise copy number events and regions using the DNACopy, and Gviz package [1].

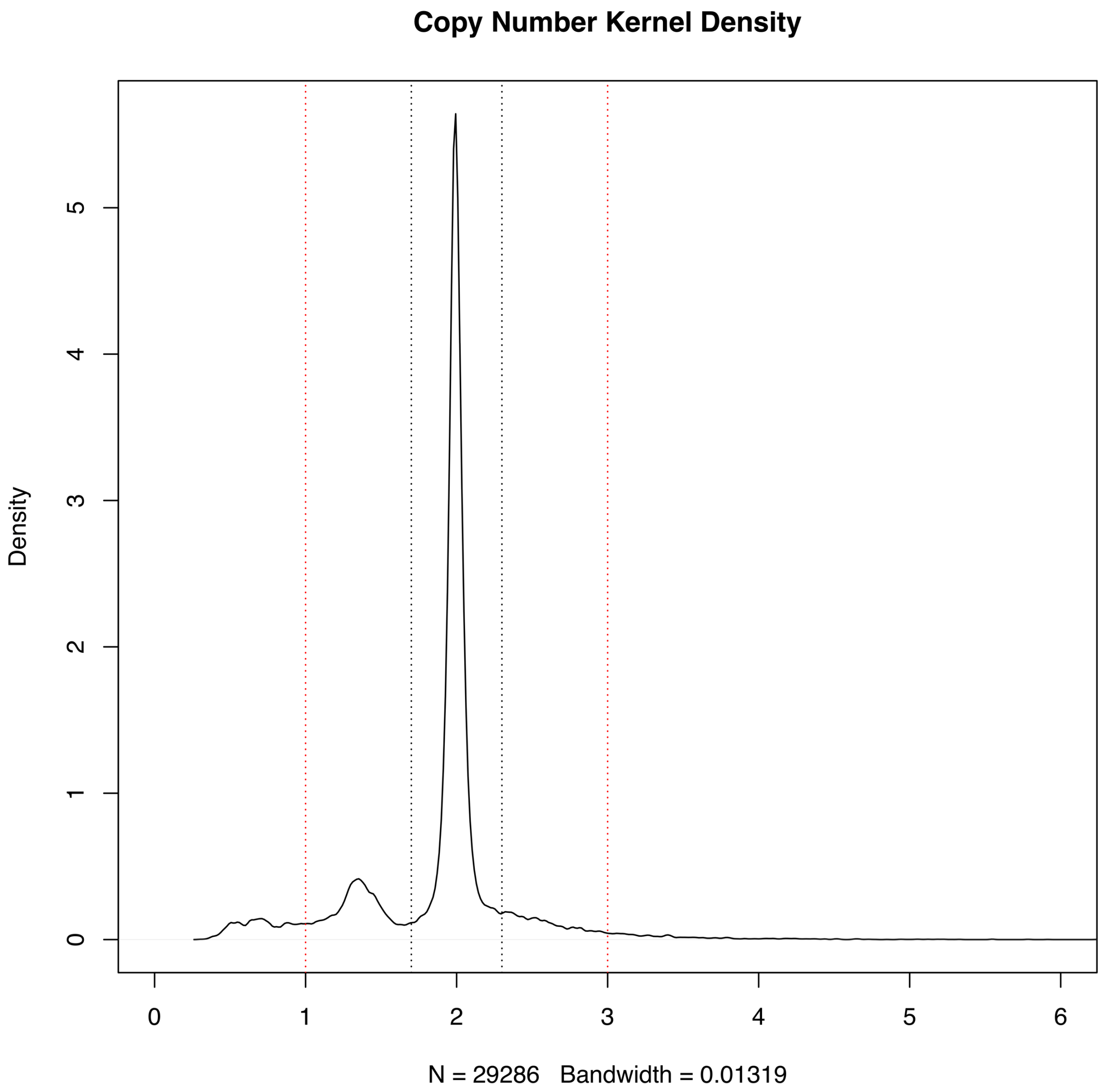


**Fig. S2. Kernel density plot of copy number values from all called segments in study.**

Figure shows the overwhelming majority of segments called by the circular binary segmentation (CBS) algorithm cluster tightly around a copy number value of two (diploid), with the median value being 1.98 (SD 0.51). Our cut-offs for calling normal copy lie either side of the peak of data > 1.7 and < 2.3 (black dotted line). The cut-offs for amplified > 3 and deleted < 1 are shown in a red dotted line.

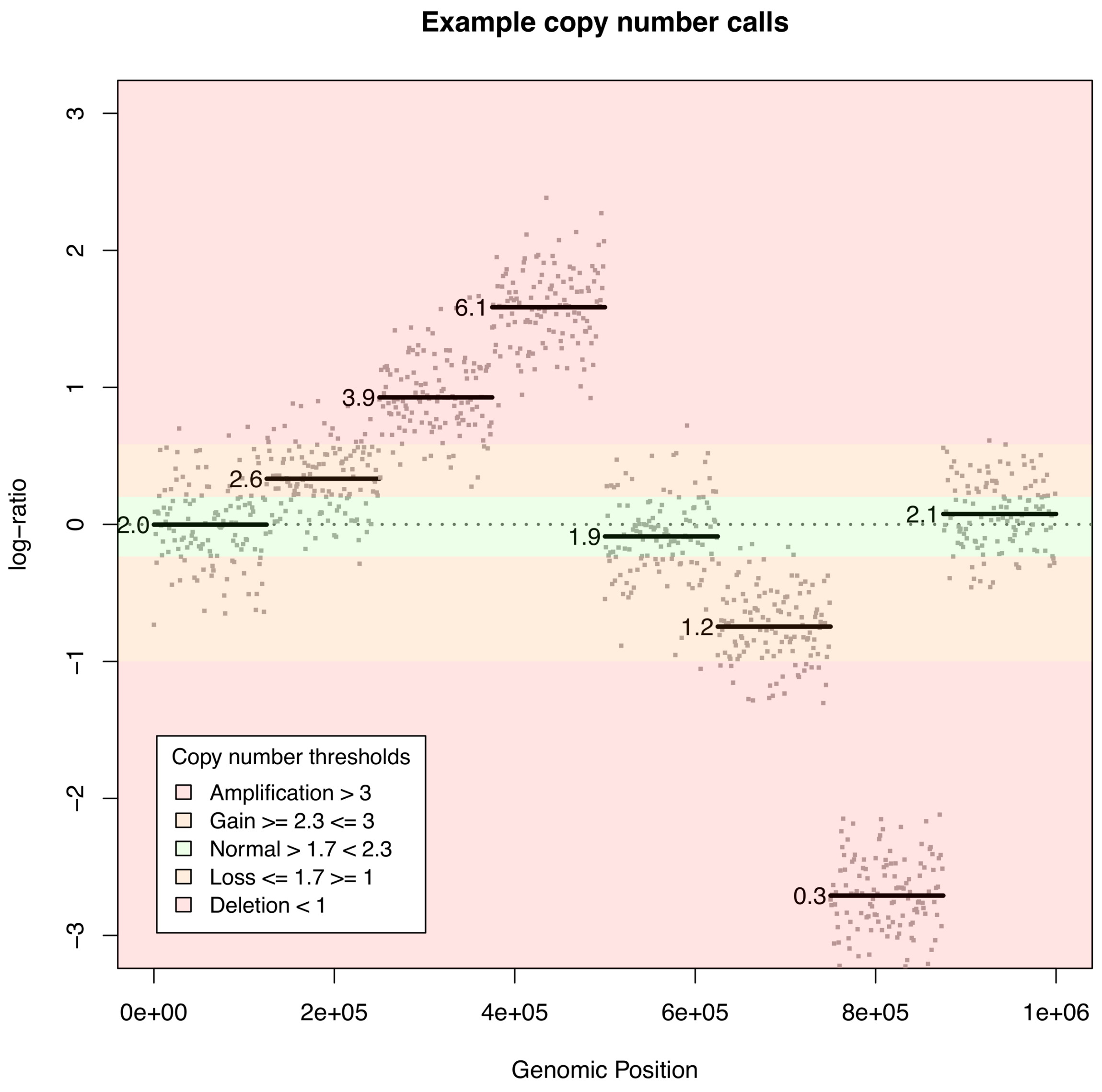


**Fig. S3. Example copy number calls showing thresholds used for amplification, gain, deletion and loss**.

Figure shows idealised example copy number calls using simulated data, grey data points represent Log *R* ratio (LRR) values for each probe, black lines represent the segmented mean LRR value, with the actual real copy number value labelled alongside. Note that conversion of copy number from $\log_2$ LRR ratios is obtained by taking the product of two raised to the power of the LRR value, and multiplying by two, $(2^{LRR})x2$, this means that deletions tend to be exaggerated in plots in comparisons to amplifications as the plot

illustrates. For this reason, we have chosen our thresholds around real values of copy rather than $\log_2$ LRR values as these are not symmetrical. Values of copy plotted with simulated LRR data in the examples have been chosen to illustrate typical: normal, gain, amplification, amplification (higher), normal, loss, deletion, and normal values of copy respectively.

## References


1. Hahne, F. & Ivanek, R. Visualizing Genomic Data Using Gviz and Bioconductor. *Methods Mol. Biol.* **1418,** 335–351 (2016).
2. Russell, L. J. *et al.* Deregulated expression of cytokine receptor gene, CRLF2, is involved in lymphoid transformation in B-cell precursor acute lymphoblastic leukemia. *Blood* **114,** 2688–2698 (2009).
3. Russell, L. J. *et al.* Characterisation of the genomic landscape of CRLF2-rearranged acute lymphoblastic leukemia. *Genes, Chromosomes and Cancer* **56,** 363–372 (2017).
4. Harvey, R. C. *et al.* Rearrangement of CRLF2 is associated with mutation of JAK kinases, alteration of IKZF1, Hispanic/Latino ethnicity, and a poor outcome in pediatric B-progenitor acute lymphoblastic leukemia. *Blood* **115,** 5312–5321 (2010).
5. Kent, W. J. BLAT--the BLAST-like alignment tool. *Genome Research* **12,** 656–664 (2002).
6. Wang, K. *et al.* PennCNV: An integrated hidden Markov model designed for high-resolution copy number variation detection in whole-genome SNP genotyping data. *Genome Research* **17,** 1665–1674 (2007).
7. Diskin, S. J. *et al.* Adjustment of genomic waves in signal intensities from whole-genome SNP genotyping platforms. *Nucleic Acids Research* **36,** e126–e126 (2008).
8. Venkatraman, E. S. & Olshen, A. B. A faster circular binary segmentation algorithm for the analysis of array CGH data. *Bioinformatics* **23,** 657–663 (2007).
9. Gentleman, R. C. *et al.* Bioconductor: open software development for computational biology and bioinformatics. *Genome Biology* **5,** R80 (2004).
10. Olshen, A. B., Venkatraman, E. S., Lucito, R. & Wigler, M. Circular binary segmentation for the analysis of array-based DNA copy number data. *Biostatistics* **5,** 557–572 (2004).
11. R Core Team. R: A language and environment for statistical computing. (2013).
